# Evolution of female multiple mating: A quantitative model of the “sexually selected sperm” hypothesis

**DOI:** 10.1111/evo.12550

**Published:** 2014-11-28

**Authors:** Greta Bocedi, Jane M Reid

**Affiliations:** 1 Institute of Biological and Environmental Sciences, University of Aberdeen, Zoology BuildingTillydrone Avenue, Aberdeen, AB24 2TZ, United Kingdom

**Keywords:** Fertilization efficiency, genetic covariance, indirect selection, polyandry, runaway coevolution, sperm competition, sexy-son

## Abstract

Explaining the evolution and maintenance of polyandry remains a key challenge in evolutionary ecology. One appealing explanation is the sexually selected sperm (SSS) hypothesis, which proposes that polyandry evolves due to indirect selection stemming from positive genetic covariance with male fertilization efficiency, and hence with a male's success in postcopulatory competition for paternity. However, the SSS hypothesis relies on verbal analogy with “sexy-son” models explaining coevolution of female preferences for male displays, and explicit models that validate the basic SSS principle are surprisingly lacking. We developed analogous genetically explicit individual-based models describing the SSS and “sexy-son” processes. We show that the analogy between the two is only partly valid, such that the genetic correlation arising between polyandry and fertilization efficiency is generally smaller than that arising between preference and display, resulting in less reliable coevolution. Importantly, indirect selection was too weak to cause polyandry to evolve in the presence of negative direct selection. Negatively biased mutations on fertilization efficiency did not generally rescue runaway evolution of polyandry unless realized fertilization was highly skewed toward a single male, and coevolution was even weaker given random mating order effects on fertilization. Our models suggest that the SSS process is, on its own, unlikely to generally explain the evolution of polyandry.

Female mating with multiple males within a single reproductive event is a widespread form of polyandry that has profound evolutionary consequences (Pizzari and Wedell 2013; Taylor et al. 2014). Such polyandry creates the opportunity for postcopulatory sexual selection and thereby drives the evolution of traits mediating sperm competition and cryptic female choice (Parker 1970; Kvarnemo and Simmons 2013; Parker and Birkhead 2013). Such polyandry might also alter the magnitudes of sexual conflict and mutation load and thereby affect population persistence (Holman and Kokko 2013). However, the fundamental questions of why such polyandry evolves and how it is maintained remain much debated (Simmons 2005; Evans and Simmons 2008; Slatyer et al. 2012; Parker and Birkhead 2013; Pizzari and Wedell 2013). Although it appears straightforward to understand that males can increase their reproductive success by mating with multiple females, the components of selection that drive the evolution of female multiple mating are often considerably less obvious (Bateman 1948; Arnold and Duvall 1994; Parker and Birkhead 2013).

In some systems, for example, where males provide nuptial gifts, multiple mating can increase female fecundity, implying that polyandry is under positive direct selection (e.g., Arnqvist and Nilsson 2000; Fedorka and Mousseau 2002; Engqvist 2007; Alonzo and Pizzari 2010; Slatyer et al. 2012). However, in other cases polyandry seems more likely to experience negative direct selection (i.e., to be costly for females), for example, because it causes physiological harm or increases disease or predation risk (e.g., Rowe 1994; Thrall et al. 2000; Chapman et al. 2003; Orsetti and Rutowski 2003; Wigby and Chapman 2005), but provides no obvious direct fitness benefit. A key remaining challenge, therefore, is to explain why polyandry evolves or is maintained in systems where direct natural selection on multiple mating seems most likely to be negative (Byrne and Roberts 2000; Hosken et al. 2003; Arnqvist and Kirkpatrick 2005; Fisher et al. 2006; Sardell et al. 2012).

Many hypotheses that invoke different forms of indirect selection, inbreeding avoidance, bet-hedging, and fertility assurance, have been proposed (Halliday and Arnold 1987; Harvey and May 1989; Keller and Reeve 1995; Zeh and Zeh 1996, 2008; Yasui 1997, 2001; Petrie and Kempenaers 1998; Jennions and Petrie 2000; Cornell and Tregenza 2007; Kokko and Mappes 2013). Prominent among these is the broad hypothesis that polyandry evolves due to indirect selection, defined as selection stemming from genetic covariances between polyandry and other female or male traits that experience direct selection (Lynch and Walsh 1998; Jennions and Petrie 2000; Simmons 2005; Pizzari and Gardner 2012; Parker and Birkhead 2013). One particularly influential suggestion is the “sexually selected sperm” hypothesis (hereafter SSS hypothesis; Harvey and May 1989; Keller and Reeve 1995). This hypothesis states that if alleles underlying polyandry are initially present at low frequency, the resulting occasional multiple mating creates opportunity for postcopulatory male–male competition to fertilize polyandrous females’ eggs. Polyandrous females will therefore increase the probability that their eggs will be fertilized by a male with relatively high “fertilization efficiency,” defined as a male's ability to succeed in postcopulatory competition for paternity through some form of sperm competition, displacement, or inhibition (Keller and Reeve 1995). If there is additive genetic variance in male fertilization efficiency, then positive genetic covariance between polyandry and fertilization efficiency is hypothesized to arise. Such covariance might cause alleles underlying polyandry to increase in frequency due to indirect selection stemming from positive direct selection on fertilization efficiency, thereby causing ongoing evolution of polyandry (Keller and Reeve 1995).

The SSS hypothesis assumes that there is additive genetic variance in both fertilization efficiency and polyandry (Evans and Simmons 2008; Evans and Gasparini 2013). In its purest form, it makes no further assumption of any preexisting genetic covariance between fertilization efficiency and polyandry, or between either trait and any other components of male or female fitness. The SSS hypothesis is therefore particularly appealing because it explains how the key genetic covariance that causes indirect selection on polyandry might arise as direct consequence of the competition over fertilization that polyandry itself creates. It therefore obviates the need to invoke any preexisting covariance due to pleiotropy or any other form of linkage. The SSS hypothesis has consequently attracted substantial interest and sparked empirical tests, and is widely cited as one plausible evolutionary explanation for polyandry (Bernasconi and Keller 2001; Simmons 2003; Simmons and Kotiaho 2007; Evans and Simmons 2008; Evans and Gasparini 2013; Klemme et al. 2014; McNamara et al. 2014).

However, there is a surprising paucity of explicit quantitative theory or models that examine whether the SSS mechanism is, in principle, sufficient to cause evolution of polyandry, thereby validating the SSS hypothesis. Curtsinger (1991) formulated a deterministic model with two diallelic loci that determine female tendency for polyandry and male sperm competitive ability, respectively, and showed that the conditions under which sperm competition can cause evolution of polyandry are extremely restricted. Specifically, there must be tight physical linkage between the two loci, positive linkage disequilibrium, and no direct fitness cost of polyandry. Curtsinger concluded that, given his model assumptions, sperm competition is unlikely to cause the evolution of polyandry. However, as Curtsinger (1991) noted and Keller and Reeve (1995) reiterated, Curtsinger's model has a well-known general limitation of single locus, diallelic models (Kirkpatrick 1982; Reeve 2000; Mead and Arnold 2004): the genetic variation attainable is very limited. The “more competitive” male genotype quickly fixates, thereby eliminating any linkage disequilibrium with polyandry and terminating coevolution. It follows that if polyandry has any direct fitness cost then the underlying allele is rapidly eliminated.

Keller and Reeve (1995) proposed that, in contrast to Curtsinger's (1991) conclusion, the SSS mechanism could in fact drive evolution of polyandry. They drew an analogy with well-established models explaining the evolution of female mating preferences for male display traits via Fisherian sexual selection (“sexy-son” models; Weatherhead and Robertson 1979; Kokko et al. 2006). In these models, alleles underlying preference, which might initially be rare, become positively genetically correlated with alleles underlying the preferred display trait due to inevitable assortative reproduction. This can cause “runaway” evolution of display away from its naturally selected optimum, causing correlated evolution of preference due to indirect selection (Fisher 1915). Since the first quantitative model that demonstrated the principle of the “runaway” process (Lande 1981), multiple different models have reached similar conclusions (reviewed in Mead and Arnold 2004; Kokko et al. 2006; Kuijper et al. 2012). When there is direct selection against the display trait but not against preference, coevolution depends on the genetic covariance between the two (*C_pd_*) and on the additive genetic variance in display (*V_d_*). Traits coevolve along lines of equilibrium with slope equal to *C_pd_*/*V_d_* (Lande 1981). Runaway coevolution is predicted if *C_pd_* > *V_d_*, whereas if *C_pd_* < *V_d_* the display only evolves to match the preference (Mead and Arnold 2004; Kokko et al. 2006). However, these lines of equilibrium are structurally unstable. When there is any direct selection against preference (i.e., preference is costly), the equilibrium is broken and preference, and the consequent coevolution of display, are eliminated (Pomiankowski 1987b; Bulmer 1989b; Barton and Turelli 1991). A variety of mechanisms, such as negatively biased mutations on the display (Pomiankowski et al. 1991), spatial variation in display (Day 2000), “condition-dependence” (Rowe and Houle 1996; van Doorn and Weissing 2004), dispersal, and negative pleiotropy (Jennions and Petrie 2000), can potentially maintain sufficient additive genetic variance in the display to rescue indirect selection and hence maintain preference even when it is costly.

Keller and Reeve (1995) suggested that female tendency for polyandry and male fertilization efficiency can be considered analogous to preference and display, respectively. They suggested that positive linkage disequilibrium between alleles underlying polyandry and fertilization efficiency will arise in exactly the same way as linkage disequilibrium between preference and display, thereby causing coevolution. They argued that the same mechanisms that might maintain genetic variation in display (e.g., negatively biased mutations) might also maintain genetic variation in fertilization efficiency and thus maintain indirect selection on polyandry even when it is costly.

However, while the verbal analogy between the “sexy-son” and the SSS processes seems compelling, at closer examination it is not necessarily perfect. Preference and display are directly linked through assortative reproduction stemming from active female choice. This is not the case for polyandry where females actively mate multiply but, at least under the pure SSS hypothesis, are assumed not to directly choose males based on their fertilization efficiency. The stochasticity introduced by precopulatory mate choice unrelated to males’ fertilization efficiency might mean that any covariance arising between polyandry and fertilization efficiency is weaker than that arising between preference and display, thereby weakening indirect selection on polyandry. Moreover, other factors that influence the outcome of postcopulatory competition for paternity, including variation in the probability of fertilization associated with mating order (e.g., first or last male precedence) or mating frequency (Birkhead and Hunter 1990; Harshman and Clark 1998; Pischedda and Rice 2012), and the relationship between males’ relative fertilization efficiency and realized paternity share, might further prevent positive linkage disequilibrium between polyandry and fertilization efficiency from developing. Because of such intrinsic biological differences between female preference for male displays versus polyandry in relation to male fertilization efficiency, there is an as yet unfulfilled need for quantitative models that explicitly validate Keller and Reeve's (1995) SSS hypothesis and identify any restrictive conditions under which it might be expected to operate.

In summary, we are left with the questions of whether polyandry could, in principle, evolve via pure Fisherian indirect selection resulting from postcopulatory competition for paternity (Harvey and May 1989; Keller and Reeve 1995) as is widely hypothesized (Simmons 2005; Evans and Simmons 2008; Klemme et al. 2014; McNamara et al. 2014), or whether such evolution requires further conditions. To answer these questions we build and compare two analogous models: one considering coevolution of preference and display and one considering coevolution of polyandry and fertilization efficiency. We thereby formalize Keller and Reeve's (1995) verbal analogy between the “sexy-son” and SSS processes and test (1) the basic premise that runaway coevolution of polyandry and fertilization efficiency can in principle occur in an analogous fashion to runaway coevolution of preference and display in the absence of direct selection; (2) whether such coevolution can continue in the presence of negative direct selection (i.e., when both traits are costly); (3) whether negatively biased mutations on fertilization efficiency can rescue indirect selection on polyandry; (4) whether coevolution between polyandry and fertilization efficiency depends on the relationship between males’ relative fertilization efficiency and realized fertilization success; (5) whether coevolution still occurs when there is environmental variance in fertilization efficiency conceptualized as mating order effects on paternity (i.e., male precedence). We thereby identify conceptual similarities and differences between the “sexy-son” and SSS processes, and discuss the degree to which the latter process might potentially drive the evolution of polyandry in nature.

## Methods

We used genetically explicit, individual-based models (e.g., Reeve 2000; Lorch et al. 2003; van Doorn and Weissing 2004; Fawcett et al. 2007; Kuijper et al. 2012) to compare coevolution of preference and display (hereafter “mate-choice model”) with coevolution of polyandry and fertilization efficiency (hereafter “SSS model”). The mate-choice model is built to recreate well-known results (Lande 1981; Pomiankowski et al. 1991; Mead and Arnold 2004; Kokko et al. 2006; Kuijper et al. 2012) within the same framework as our new SSS model, facilitating comparison between the “sexy-son” and SSS processes. The two models are analogous in every respect except for differences stemming from key biological characteristics of the different traits considered. Individual-based models do not require restrictive a priori assumptions regarding the distributions of genotypic values or the magnitude or direction of genetic covariances. Rather, they can allow these distributions and covariances to emerge, facilitating investigation of the magnitudes and dynamics of arising linkage disequilibria.

### Genetic Architecture

Both models assume a diploid additive genetic system with two autosomal traits: female preference (*P*) and male display (*D*) in the mate-choice model, and female tendency for polyandry (*Py*) and male fertilization efficiency (*F*) in the SSS model. In each model, all individuals of both sexes carry *L* diploid physically unlinked loci underlying both traits, with sex-limited phenotypic expression. Any genetic covariance between female and male traits therefore results exclusively from linkage disequilibrium generated by assortative reproduction. We assumed a continuum-of-alleles model (Kimura 1965; Lande 1976; Reeve 2000) whereby the possible allelic distribution at each locus comprises an infinite number of alleles producing a continuous distribution of genetic effects. Each individual's genotypic value is calculated as the sum of all 2*L* allelic values underlying each trait (hereafter denoted *gP*, *gD*, *gPy*, and *gF* for the four traits, respectively). We did not model any explicit environmental variance, meaning that individuals’ phenotypic values (hereafter *P*, *D*, *Py*, and *F*) are primarily determined by their genotypes. However, because of stochastic processes affecting trait expression (see below), realized heritability is in some cases less than one.

Models examining mate choice evolution typically allow preference and display traits to assume any real number (e.g., Lande 1981; Pomiankowski et al. 1991; Pomiankowski and Iwasa 1998; Higashi et al. 1999; Day 2000). Although this is mathematically convenient and justifiable in some cases, allowing negative values does not make biological sense for polyandry because females can mate once or more times, but cannot mate a negative number of times. We therefore allowed the male phenotypes (*D* and *F*) to take any real value, but constrained the female phenotypes (*P* and *Py*) to be equal to or greater than zero by equating any negative genotypic value to zero. Preliminary simulations for our mate-choice model that did and did not constrain female phenotypes to be positive both quantitatively reproduced expectations from previous theory (Lande 1981; Mead and Arnold 2004; Kokko et al. 2006). Our constrained model is therefore appropriate to test the SSS hypothesis in relation to existing models of mate choice.

For each locus, offspring inherit single random alleles from their mother and father. Each allele has a mutation probability of μ per generation. When a mutation occurs, a mutational effect sampled from a normal distribution with mean *m* and variance σ*^2^_m_* [i.e., *N*(*m, σ^2^_m_*)] is added to the allele value (Table S1; Kimura 1965; Lande 1976). Negatively biased mutational effects on male traits are sampled from a normal distribution *N*(*m*′, σ*^2^_m_*) (Table S1).

### Mating, Reproduction, and Survival

We modeled a single, freely mixing population with nonoverlapping generations, and a 1:1 primary sex ratio. Each generation starts with reproduction, split into mating, fertilization, and birth stages. After reproduction, all adults die and offspring survive to adulthood according to density dependence and viability selection.

The mating phase differs conceptually between the mate-choice and SSS models. In the mate-choice model, each female chooses a male according to the strength of her preference and sampled male displays. We implemented a best-of-N model (Seger 1985; Fawcett et al. 2007), where the female chooses from a random sample of *Nmales_I_* males. This constraint facilitates analogy with the SSS model, where polyandrous females typically mate with relatively few males randomly sampled from the population (see below). The best-of-N constraint introduces stochasticity into precopulatory mate choice, causing the heritability of preference to be less than one and reducing the correlation between preference and display (Benton and Evans 1998). This model is therefore conservative regarding possible runaway coevolution. Within the random sample of males, each male *j* has a probability (*pc_j_*) of being chosen by female *i* given by (Lande 1981; Pomiankowski et al. 1991; Higashi et al. 1999; Lorch et al. 2003; Fawcett et al. 2007):

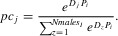
(1)

To avoid numerical errors, we imposed an arbitrary maximum phenotypic value of 60 for *D*, thereby curtailing ongoing runaway toward infinite values. Imposing this maximum does not hinder initial coevolution. Each female produces a number of offspring sampled from a Poisson distribution with mean *R* and all offspring are sired by the female's chosen male. Individual males can mate with unlimited females.

In the SSS model we assumed that males cannot directly advertise their fertilization efficiency, meaning that females cannot exert direct precopulatory choice (therefore conceptualizing the pure SSS hypothesis; Keller and Reeve 1995). Each female mates with a number of randomly selected males, *Nmales_II_*, given by

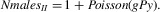
(2)

All females therefore mate at least once (which is assumed to ensure full fertility), and the number of additional matings is positively correlated with a female's genotypic value for polyandry. This formulation relaxes the unrealistic assumption that polyandrous females mate with as many males as necessary to sample the population's entire sperm pool (as assumed by Curtsinger 1991). One assumption of the mate-choice model, that preference *P* is continuously distributed (e.g., Lande 1981; Pomiankowski et al. 1991; Pomiankowski and Iwasa 1998; Higashi et al. 1999; Day 2000), is not appropriate for polyandry *Py* because females mate a discrete number of times. Equation (2) translates continuous genetic variation in *gPy* into a discrete phenotype *Py*. This introduces some additional nongenetic variance in *Py* compared to *P*, constituting a conceptual difference between the mate-choice and SSS models. Using threshold models to translate continuous variation in *gPy* to discrete variation in *Py* yielded similar conclusions.

As in the mate-choice model, each female produces a number of offspring sampled from a Poisson distribution with mean *R*. After mating, all the female's mates compete for the paternity of each offspring (conceptually, through sperm competition and related mechanisms, Parker 1970; Keller and Reeve 1995). Some function relating each male's fertilization efficiency *F* to realized fertilization success and paternity is therefore required. Our primary model assumes a “fair-raffle” (Parker 1990) weighted by each male's relative *F*. The probability, *pf_j_*, for male *j* to fertilize each of female's eggs is:

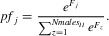
(3)

As for equation (1), we imposed an arbitrary maximum value of 60 for *F*. We used equation (3) for fertilization probability to facilitate analogy with the probability that a male would be chosen in relation to his display in the mate-choice model (eq. 1; Lande 1981). However, the two functions differ, reflecting an intrinsic difference between the pre- and postcopulatory processes. Specifically, while *P* and *D* are directly linked through precopulatory female choice, *Py* and *F* are only indirectly linked via the postcopulatory competition for paternity created by polyandry. Female preference is part of equation (1) because females exert direct choice among sampled males. By contrast, polyandry does not appear in equation (3) because females do not directly choose males based on their fertilization efficiency.

The offspring survival phase is identical in both models. Each offspring, *i*, has a survival probability, *ps_i_*, determined by the population carrying capacity *K* and its individual viability, *v_i_*, weighted by the sum of the viabilities of all offspring (*N_off_*) in the population (cf. Burton et al. 2010):

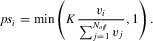
(4)

When no direct fitness cost is applied to any of the four focal traits, *v_i_* = 1. Costs are applied through stabilizing selection toward a naturally selected optimum, θ, for the trait (Haldane 1954; Lande 1981; Bulmer 1989a). Generally, given nonzero cost of trait *t*, individual viability is:

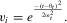
(5)

Here, *t* represents the phenotypic value of the trait (*D* or *F* for males and *P* or *Poisson*(*gPy*) for females), θ*_t_* the trait's naturally selected optimum and ω*_t_* the strength of stabilizing selection, where higher values of ω*_t_* correspond to weaker selection (see Fig. S1).

### Form of Fertilization

Fair-raffle fertilization, where males that mate with a particular female fertilize ova in approximate proportion to their relative *F* values, may be common in nature (Simmons 2014, see Discussion). However there are other conceivable outcomes, such as a “winner-takes-all” scenario where the male with the highest *F* fertilizes all of a female's ova irrespective of the relative *F* values of the female's other mates (e.g., Simmons and Siva-Jothy 1998; Wedell and Cook 1998; Simmons 2014). This scenario is conceptually more similar to female preference for male display, where all of a female's offspring are sired by the single preferred male. The operation of the SSS process might therefore depend on the relationship between a male's relative *F* and his realized fertilization success. To investigate this dependence we modified (3) as:

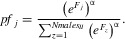
(6)

Here, α determines the degree to which postcopulatory fertilization success is biased toward the male with the highest *F* value out of each female's mates. A value of α = 1 corresponds to the fair-raffle weighted by relative *F* (eq. 3), whereas α > 1 biases fertilization success more strongly toward the male with the highest *F* up to “winner-takes-all,” whereas α < 1 weakens the association between *F* and fertilization success, such that paternity is distributed more evenly than in the *F*-weighted fair-raffle.

### Male Precedence

In nature, strong first or last male precedence is often observed, where the first or last male to mate with a female sires a disproportionate number of offspring (e.g., Parker 1970; Birkhead and Hunter 1990; Watson 1991; Lacey et al. 1997; Price et al. 1999; Kraaijeveld-Smit et al. 2002). When mating order is not itself genetically determined, male precedence implies strong environmental modulation of underlying genetic effects on fertilization. We therefore further investigated the degree to which such environmental effects and resulting precedence could modulate the SSS process. Here, a male's fertilization probability is determined not only by his *F* value but also by the rank order in which he mates with the female relative to her other mates, assuming first male precedence. Because mating order is random in our model, the results would be identical assuming last male precedence. The probability, *pf_j_*, for male *j* to fertilize each of female's eggs becomes:

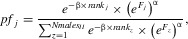
(7)
where β represents the strength of precedence and *rank_j_* is the rank order with which male *j* mated with female *i* (0 ≤ *rank_j_* ≤ *Nmales_II_*; see Fig. S2).

### Simulations

We ran simulations that quantified coevolution of male and female traits (*D* and *P* in the mate-choice model and *F* and *Py* in the SSS model) with (1) no direct selection on either trait (i.e., no costs); (2) direct selection on both traits (i.e., both are costly); (3) negatively biased mutations on the male trait; (4) different relationships between relative *F* and realized fertilization; (5) nongenetic precedence in fertilization. When direct selection was applied, we varied the strength of selection, ω, on the female traits, but held selection on the male traits constant (ω*^2^_D_ = ω^2^_F_* = 1.0). Conclusions remained similar when selection on the male traits was also varied. All model variables and parameter values are summarized in Table S1. Five hundred females and 500 males were initialized in each simulation. Initial allelic values for each trait were sampled from specified normal distributions (Table S1; Fig. S3). Thereafter, the distributions of genetic and phenotypic values for the four traits, and associated genetic (co)variances, emerged from the processes of drift, selection, and mutation encapsulated in the model and were otherwise unconstrained. All simulations were run for 10,000 generations, checked for equilibrium, and replicated 50 times. We report results for the first 5000 generations as all simulations had already equilibrated. We primarily present cross-sex genetic correlations instead of covariances to allow comparison between models (as correlations are unit-free variance-standardized covariances). Genetic variances and covariances are provided as Supporting Information. Genetic correlations and covariances arising within each generation of each simulated population were calculated across the within-individual genotypic values of male and female traits.

To quantify female trait values at mutation-selection balance, we additionally simulated “neutral” traits with no function (meaning that each female mated with one random male) but the same cost as the active female traits (i.e., *P* or *Py*). Finally, we quantified model sensitivity to the number of loci *L*, the mutation rate μ, and the mutational variance σ*^2^_m_*. Conclusions were not sensitive to the value of *R*.

## Results

### Basic Models

**Figure 1:**
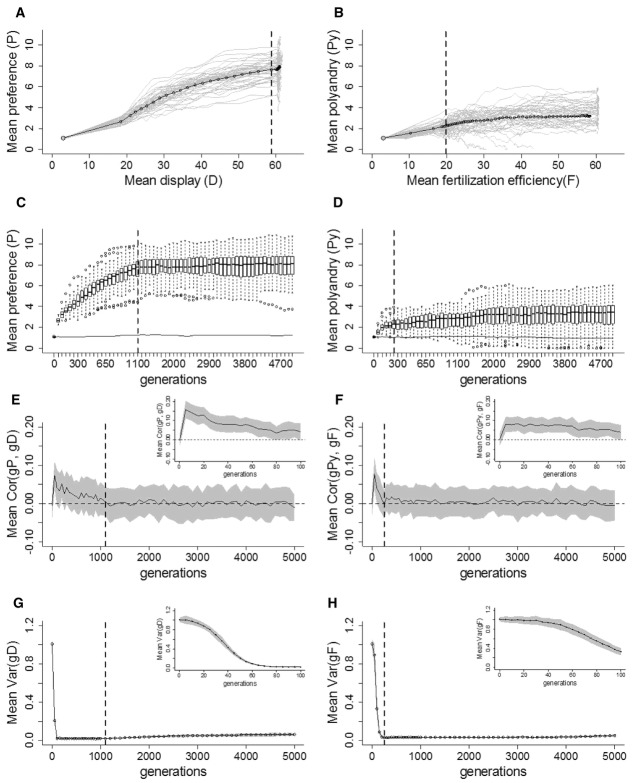
Coevolution of preference (*P*) and display (*D*; A, C, E, G), and of polyandry (*Py*) and fertilization efficiency (*F*; B, D, F, H), in the absence of costs. (A and B) Overall mean phenotypic trait values (black lines), mean values for individual replicate simulations (gray lines), and simulation starting values (gray circles) for (A) preference and display, and (B) polyandry and fertilization efficiency. (C and D) Evolutionary trajectories of *P* and *Py* across generations described as medians (black bands), first and third quartiles (box limits), and approximately twice the standard deviation (whiskers). Black lines show the mean values of “neutral” traits subject only to mutation. (E and F) Mean correlation (black lines) ± standard deviation (gray shading) between the genotypic values for preference, *gP*, and display, *gD* [Cor(*gP*,*gD*)]; and for polyandry, *gPy*, and fertilization efficiency, *gF* [Cor(*gPy*,*gF*)]. Dashed horizontal lines demarcate zero correlation. (G and H) Mean genotypic variances in *D* and *F* (black lines) ± SD (gray shading). (E–H) Inserts: Same correlations and variances in the first 100 generations at five generation intervals. All means are averaged over 50 replicate simulations and plotted every 50 generations until generation 1000 and 100 generations thereafter. In all plots, dashed vertical lines indicate when the mean genetic correlation went to zero.

When there was no direct selection on preference or display (i.e., both traits were cost-free), the mate-choice model produced the expected Fisherian “runaway,” where both traits coevolved and increased in value across generations (up to the imposed numerical limit; Fig. 1A, C). Runaway coevolution occurred consistently across replicate simulations (Fig. 1A, C).

The basic SSS model, where polyandry and fertilization efficiency were cost-free with “fair-raffle” fertilization, produced qualitatively weaker coevolution (Fig. 1B, D). Generally, mean *Py* slowly increased or persisted around the initialization value while mean *F* increased. However, in some replicates polyandry did not evolve at all, but instead decreased to zero (Fig. 1B, D).

In both models, the expected positive genetic correlation between the female and male traits arose in the first few generations, but was larger in the mate-choice model than in the SSS model (Fig. 1E, F). The correlations peaked within the first five generations then gradually decreased to zero. The correlation between *gP* and *gD* decreased to zero only once the “runaway” was halted by the artificial maximum imposed on *D* (Fig. 1). However, the correlation between *gPy* and *gF* decreased to zero well before *F* reached the imposed maximum, effectively stopping coevolution of *Py* and *F* (Fig. 1). As expected, the genotypic variance for the male traits decreased quickly in both models (Fig. 1G, H). However, it decreased earlier in the mate-choice model, resulting from stronger sexual selection on display than on fertilization efficiency (Fig. S4).

### Direct Selection (Costs)

**Figure 2:**
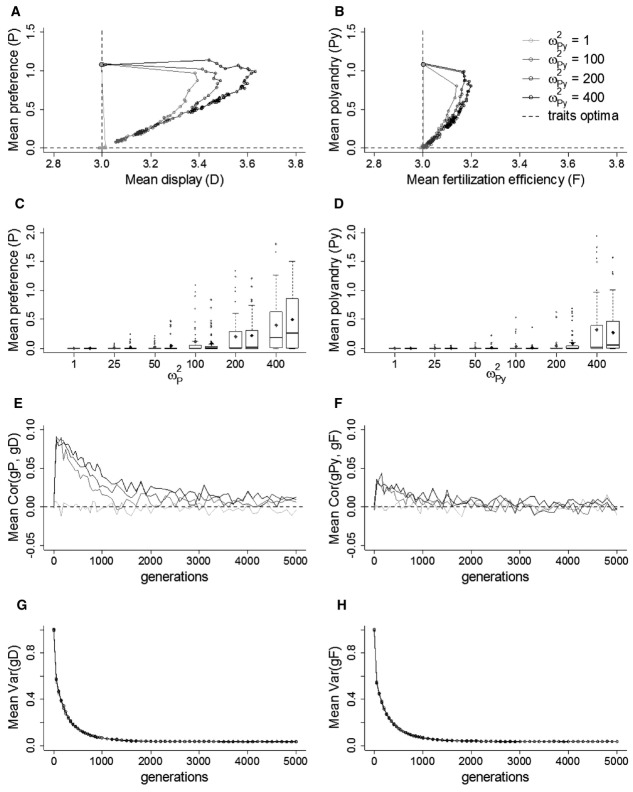
Coevolution of preference (*P*) and display (*D*; A, C, E, G), and of polyandry (*Py*) and fertilization efficiency (*F*; B, D, F, H), for selected costs. (A and B) Mean values for (A) *P* and *D*, and (B) *Py* and *F* for different strengths of direct selection on the female traits (ω*^2^_P_* and ω*^2^_Py_*). Gray circles indicate the simulation starting values. Dashed lines indicate the traits’ optima. (C and D) Phenotypic values of *P* and *Py* (white boxes) and neutral traits subject to the same strength of direct selection (gray boxes) at generation 5000, described as medians (solid bands), first and third quartiles (box limits), approximately twice the standard deviation (whiskers), and means (diamonds). (E and F) Mean genetic correlation between preference, *gP*, and display, *gD* [Cor(*gP*,*gD*)]; and between polyandry *gPy*, and fertilization efficiency, *gF* [Cor(*gPy*,*gF*); color codes as in (A and B)]. Dashed lines demarcate zero correlation. (G and H) Mean genotypic variance in *D* and *F*. All values are averaged over 50 replicates and plotted every 50 generations until generation 1000 and every 100 generations thereafter.

As expected from previous theory (Pomiankowski 1987b; Bulmer 1989b; Barton and Turelli 1991), the mate-choice model showed that when both preference and display experienced direct selection (i.e., were costly), *P* eventually stabilized at the mean value expected under mutation-selection balance (Fig. 2A, C). For small costs, the slower decrease in *P* allowed *D* to be temporarily pulled away from mutation-selection balance but, as *P* decreased, *D* gradually decreased back to mutation-selection balance.

A similar pattern emerged for polyandry and fertilization efficiency when both were costly, in that neither persisted above mutation-selection balance (Fig. 2B, D). Therefore, as for *P* and *D*, coevolution of *Py* and *F* did not readily occur in the presence of opposing direct selection.

The initial mean genetic correlation between *gPy* and *gF* was less than half that between *gP* and *gD*, especially for lower costs on the respective female traits. Generally, in both models, the higher the cost on the female trait, the smaller the initial correlation between female and male traits and the faster the correlation decreased across generations. Genotypic variances and covariances are shown in Figure S5.

### Biased Mutations

**Figure 3:**
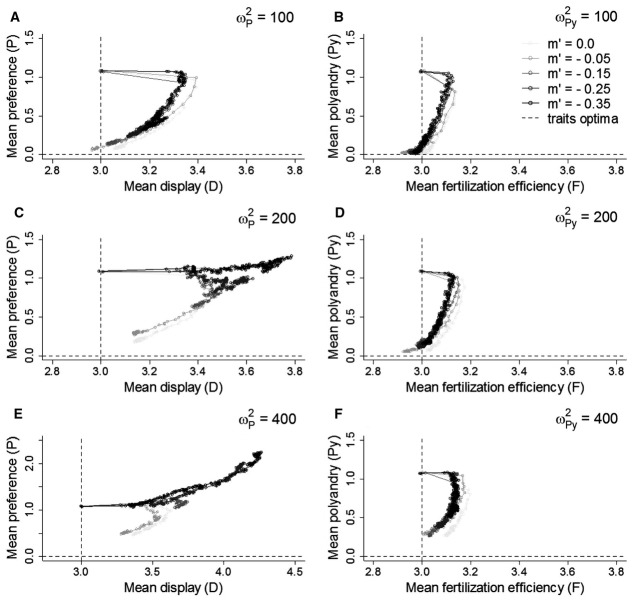
Effect of negatively biased mutations (*m*′) in the male trait on coevolution between preference (*P*) and display (*D*; A, C, E), and between polyandry (*Py*) and fertilization efficiency (*F*; B, D, F), for three different magnitudes of direct selection on *P* and *Py* (ω*^2^_P_* and ω*^2^_Py_*). Mean phenotypic values are averaged over 50 replicate simulations, plotted every 50 generations until generation 1000 and every 100 generations thereafter. Dashed lines indicate the traits’ optima.

**Figure 4:**
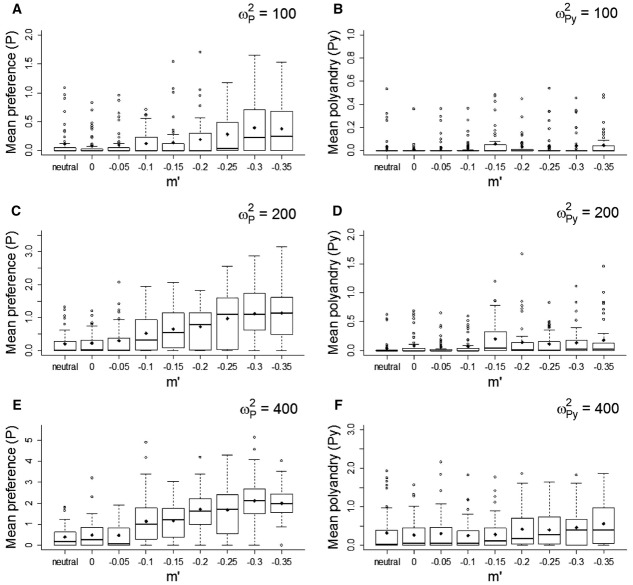
Effect of negatively biased mutations (*m*′) in the male trait on coevolution between preference (*P*) and display (*D*; A, C, E), and between polyandry (*Py*) and fertilization efficiency (*F*; B, D, F), shown through the population mean *P* and *Py* at generation 5000. Data are presented for three different levels of direct selection on *P* and *Py* (ω*^2^_P_* and ω*^2^_Py_*). Phenotypic values of these traits and neutral traits subject to the same strength of direct selection (“neutral”) are presented as medians (solid bands), first and third quartiles (box limits), approximately twice the standard deviation (whiskers), and means (diamonds) over 50 replicate simulations.

As expected (Pomiankowski et al. 1991), introducing negatively biased mutations on display *D* maintained some genetic variation and hence maintained preference away from mutation-selection balance (Figs. 3 and 4A, C, E). Stronger negatively biased mutations on *D* (i.e., more negative mean *m*′ of the distribution of mutational effects) and weaker direct selection on *P* resulted in higher equilibrium values of both *P* and *D*. The higher the cost on *P*, the stronger the negative mutation bias necessary to maintain *P* away from mutation-selection balance (Fig. 4).

In contrast, in the SSS model, imposing negatively biased mutation on fertilization efficiency failed to substantially rescue coevolution with polyandry (Figs. 3 and 4B, D, F). Even for small costs of polyandry, strong negatively biased mutations led to mean equilibrium values for *Py* that were very close to the expected mutation-selection balance (Figs. 3F and 4F).

In both models, negatively biased mutations had the expected effect of maintaining greater genetic variance in the male trait (Fig. S6). However, the magnitude of mutation bias hardly affected the magnitude of the genetic correlation between female and male traits (Fig. S7). For lower costs on the female traits, in the presence of negatively biased mutations, a slightly higher correlation was maintained between *gP* and *gD*, while the correlation between *gPy* and *gF* was not affected. Therefore, in our simulations with fair-raffle fertilization, Keller and Reeve's (1995) proposition that the SSS process, and resulting evolution of polyandry, could be rescued by negatively biased mutations in fertilization efficiency is not supported.

### Form of Fertilization

**Figure 5:**
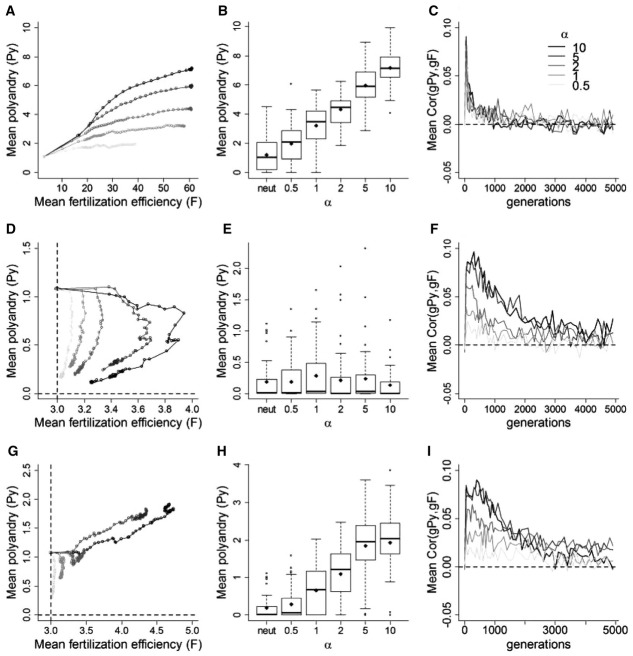
Effect of the strength of paternity bias (α) linking fertilization efficiency (*F*) to realized fertilization success on coevolution between polyandry (*Py*) and fertilization efficiency (*F*). (A) Mean phenotypic values for *Py* and *F* for different values of α in the absence of costs. α = 1 and α = 10 equate to “fair-raffle” and “winner-takes-all” fertilization, respectively. Gray circles indicate the simulation starting values. (B) Mean phenotypic values for *Py* and neutral traits (“neutral”) subject to the same strength of direct selection at generation 5000, for different values of α, described as medians (solid bands), first and third quartiles (box limits), approximately twice the standard deviation (whiskers), and means (diamonds). (C) Mean correlations between *Py* and *F* for different values of α. (D–F) Same as in (A–C) when costs are applied to both traits (ω*^2^_Py_* = 400.0 and ω*^2^_F_* = 1.0). (G–I) Same as in (D–F) when, additionally to costs, substantial negatively biased mutations are applied to *F* (*m*′ = −0.35). All data are averaged over 50 replicate simulations, plotted every 50 generations until generation 1000 and every 100 generations thereafter. Dashed lines in (D) and (G) indicate the traits’ optima.

Coevolution between polyandry and fertilization efficiency was influenced by the relationship between relative male *F* and realized fertilization success (Fig. 5). With no costs, fertilization scenarios that tended toward “winner-takes-all” (α > 1) lead to reliable runaway coevolution between *Py* and *F* (Fig. 5A, B) and the stochasticity observed with “fair-raffle” fertilization (α = 1) progressively disappeared (Fig. S8). In contrast, when paternity was shared more evenly than under an *F*-weighted fair-raffle (α < 1), coevolution was even weaker and more stochastic, and *Py* frequently decreased to zero or remained at the initialization value (Figs. 5A, B, and S8). Furthermore, even given “winner-takes-all” fertilization, coevolution did not occur when polyandry was costly; *Py* then equilibrated at mutation-selection balance (Fig. 5D, E). However, with a low cost on *Py*, strong negatively biased mutations on *F* “rescued” polyandry and maintained the population mean above mutation-selection balance (Figs. 5G, H, and S9). Indeed, the stronger the fertilization bias toward the male with the highest *F* the stronger the genetic correlation between *Py* and *F*. Genotypic variances and covariances are shown in Figure S10.

### Male Precedence

**Figure 6:**
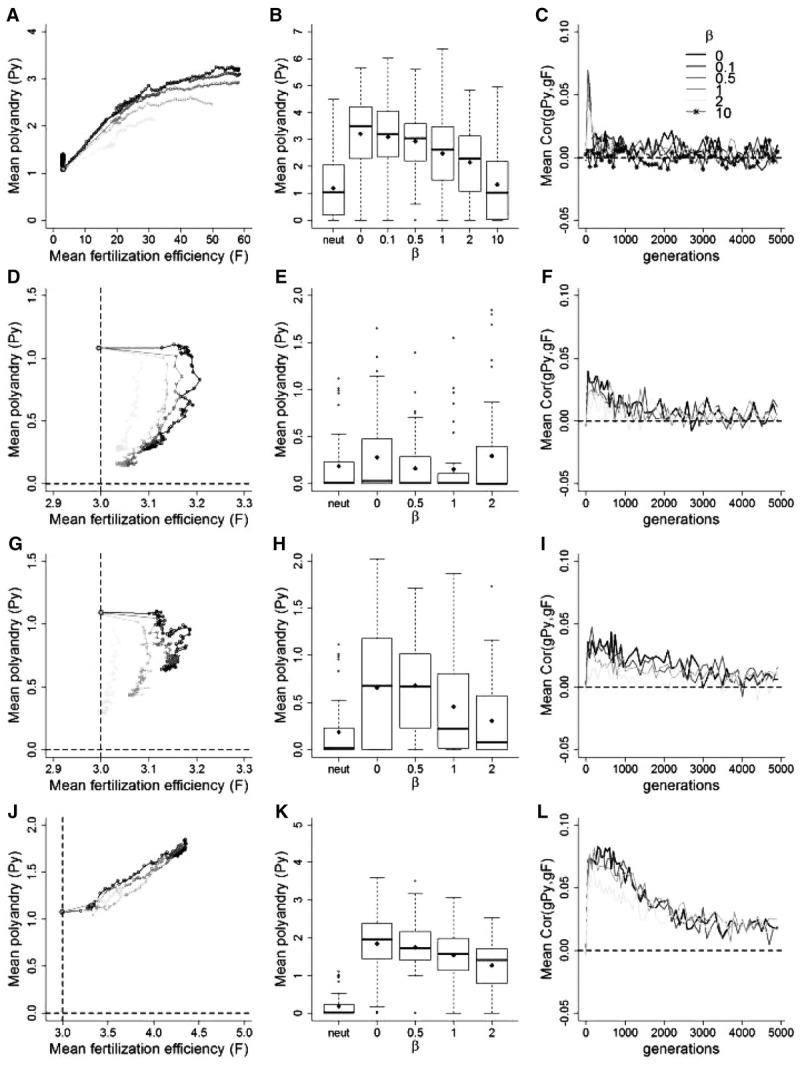
Effect of male precedence in fertilization (β) on coevolution between polyandry (*Py*) and fertilization efficiency (*F*). (A) Mean phenotypic values for *Py* and *F* for selected values of β in the absence of costs. Gray circles indicate the simulation starting values. (B) Mean phenotypic values for *Py* and neutral traits (“neutral”) subject to the same strength of direct selection at generation 5000, for different values of β, described as medians (solid bands), first and third quartiles (box limits), approximately twice the standard deviation (whiskers), and means (diamonds). (C) Mean correlations between *Py* and *F* for different values of β. (D–F) Same as in (A–C) when costs are applied to both traits (ω*^2^_Py_* = 400.0 and ω*^2^_F_* = 1.0). (G–I) Same as in (D–F) when, additionally to costs, substantial negatively biased mutations are applied to *F* (*m*′ = −0.35). (L–N) Same as in (G–I), with realized fertilization biased toward males with higher *F* (α = 5). All data are averaged over 50 replicate simulations, plotted every 50 generations until generation 1000 and every 100 generations thereafter. Dashed lines in (D) and (G) indicate the traits’ optima.

Assuming “fair-raffle” fertilization, the occurrence of nongenetic male precedence in fertilization further reduced the genetic correlation between *gPy* and *gF*, causing even weaker coevolution than in the absence of any such precedence (Fig. 6C, F, I, N). Of course, the extreme case where the first male to mate fertilizes all the female's eggs (β = 10.0), eliminated the opportunity for selection on fertilization efficiency, causing *F* (and indirectly *Py*) to be effectively neutral in absence of costs (Fig. 6A–C). Stronger precedence then caused *Py* and *F* to evolve to lower mean values than without any precedence.

When both *Py* and *F* were costly, *Py* stabilized at mutation-selection balance (Fig. 6D–F), as with no precedence. Moreover, *F* equilibrated at lower values for higher degrees of precedence (Fig. 6D). Imposing negatively biased mutations on *F* (Fig. 6G–I) did not change this general pattern. The same was true when fertilization differed from “fair-raffle” (Fig. 6L–N). With high paternity bias (α = 5), low cost and negatively biased mutations, *Py* still evolved given some precedence. However, the genetic correlation between *Py* and *F* and the equilibrium value of *Py* decreased with increasing precedence. Genotypic variances and covariances are shown in Figure S11.

### Sensitivity Analyses

**Figure 7:**
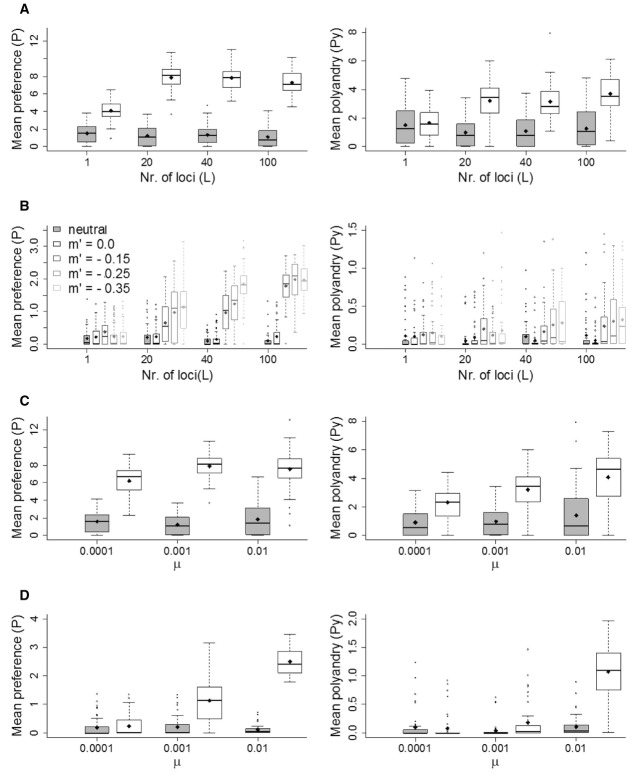
Sensitivity to the number of loci (*L*) underlying each trait's genotypic value and mutation rate (μ; mutation probability per allele per generation). (A) Mean phenotypic values for preference (*P*) and polyandry (*Py*) (white boxes), and mean values of “neutral” traits subject only to mutation (gray boxes) at generation 5000, for different values of *L* in the absence of any cost. Data are described as medians (solid bands), first and third quartiles (box limits), approximately twice the standard deviation (whiskers), and means (diamonds). (B) Same as in (A), when traits are costly (ω*^2^_P_* = ω*^2^_Py_* = 200.0), and display (*D*) and fertilization efficiency (*F*) are subject to negatively biased mutations (*m*′; see plot legend). (C) Mean phenotypic values for *P* and *Py* (white boxes), and mean values of “neutral” traits subject only to mutation (gray boxes) at generation 5000, for different mutation rates μ in the absence of any cost. (D) Same as in (C), when traits are costly (ω*^2^_P_* = ω*^2^_Py_* = 200.0) and *D* and *F* are subject to negatively biased mutations (*m*′ = −0.35). All data are averaged over 50 replicate simulations.

In the absence of costs, both the mate-choice and SSS models were sensitive to the number of loci, *L* (Figs. 7A and S12A). The main difference was between *L* = 1 and *L* > 1. With *L* = 1, the genetic variances and correlation between the female and male traits rapidly tended toward zero (Figs. S13A, B and S14A, B). This effectively stopped runaway coevolution and the female trait evolved to lower values than in corresponding multilocus models. With costs, results were independent of *L* and the female traits equilibrated at mutation-selection balance (Fig. S12B–D; cf. Fig. 2). However, the combination of multiple loci and strong negatively biased mutations on the male trait resulted in higher mean equilibrium values of *P* and *Py* (Fig. 7B).

Both models were sensitive to mutation rate, μ, in the absence of costs (Figs. 7C and S15A) and, given costs, in the presence of strong negatively biased mutations on the male trait (Figs. 7D and S15C). Given weak direct selection on polyandry (ω*^2^_Py_* = 200.0), high mutation rate (μ = 0.01), and strong negatively biased mutations (*m*′ = −0.35) polyandry was, on average, maintained at or above its initialization values (Figs. 7D and S15C), although showed considerable stochasticity (Fig. 7D). This outcome was associated with the maintenance of high genetic variance in the female and male traits and consequent higher genetic covariance (Fig. S17).

## Discussion

Following Keller and Reeve's (1995) compelling formulation, the SSS hypothesis, which proposes that polyandry (*Py*) evolves due to indirect selection stemming from positive genetic covariance with male fertilization efficiency (*F*), has been widely cited as one plausible evolutionary explanation for polyandry. However, there remains a surprising lack of quantitative models that formally validate the premise of the SSS hypothesis or identify conditions under which it might operate. We developed a genetically explicit model to test whether the SSS hypothesis is valid despite contrary conclusions drawn from simple diallelic models (Curtsinger 1991). Specifically, we considered whether indirect selection created by postcopulatory competition over fertilization could be sufficient to promote evolution of polyandry in an analogous way to the evolution of female preferences (*P*) through positive genetic covariance with male displays (*D*). We conclude that while it is possible, under certain conditions, for intrinsic genetic covariances to arise and cause or maintain some level of polyandry, these conditions are rather restricted. In the presence of even weak direct selection against polyandry (i.e., small costs), it appears unlikely that polyandry could generally evolve or be maintained exclusively due to intrinsic genetic covariance with fertilization efficiency resulting from assortative reproduction.

### Evolution of Cost-Free Polyandry

In the absence of direct selection against polyandry and fertilization efficiency, polyandry did evolve due to intrinsic indirect selection as postulated (Harvey and May 1989; Keller and Reeve 1995). Such evolution occurred consistently when fertilization tended toward “winner-takes-all,” such that all of a female's offspring were sired by the male with whom she mated that had the highest *F*. However, when fertilization followed a “fair-raffle” weighted by the relative *F* values of each female's mates, evolution of polyandry was less reliable than evolution of female preference. The stochastic outcome of the SSS process under the “fair-raffle,” and the overall dependency on the relationship between relative *F* and realized fertilization success, suggests that the SSS process might not on its own provide a deterministic explanation for the widespread evolution of polyandry in nature, even when polyandry and high fertilization efficiency are both completely cost-free.

With “fair-raffle” fertilization, the emergent genetic correlation between *Py* and *F* was weaker than the analogous genetic correlation between *P* and *D*. The analogy between the “sexy-son” and SSS processes, on which the SSS hypothesis relies (Keller and Reeve 1995), is then only partly valid. This is due to inherent differences between the assumed biological processes of precopulatory female preference and postcopulatory male competition for fertilization. Key is the division of paternity across females’ offspring, which influences the reproductive and genetic associations between the female and male traits. With female preference, the preferred male is assumed to sire all the female's offspring, creating a direct reproductive and genetic association between *P* and *D*. By contrast, assuming that males cannot advertise their fertilization efficiency and “fair-raffle” fertilization, paternity is shared between the female's mates proportionally to their *F* values. Males with high *F* are then likely to sire at least some offspring of polyandrous females, thereby creating some positive genetic covariance between *Py* and *F*. However, as genetic variance in *F* is eroded paternity will likely be shared among the female's mates, thus reducing any genetic covariance between *Py* and *F*. Coevolution between polyandry and fertilization efficiency stemming from the SSS process is then weaker than the analogous coevolution between preference and display predicted by long-standing models of Fisherian runaway. When paternity is disproportionately biased toward males with higher *F*, the SSS process becomes more similar to the “sexy-son” process.

### Direct Selection and Negatively Biased Mutations

The SSS hypothesis seems appealing because it suggests one means by which positive indirect selection on polyandry might arise, thereby explaining why polyandry might evolve and persist despite negative direct selection (Simmons and Kotiaho 2007; Evans and Simmons 2008; Evans and Gasparini 2013; McNamara et al. 2014). However, this logic is clearly invalid if the SSS mechanism that is postulated to cause positive indirect selection on polyandry does not work when there is negative direct selection (i.e., when polyandry is costly). Through their basic model formulations, Curtsinger (1991) and Keller and Reeve (1995) both concluded that the basic SSS process cannot work given any direct cost of polyandry. However, this point is rarely mentioned in subsequent literature, and the SSS hypothesis is often (inconsistently) invoked as an explanation for ongoing evolution or maintenance of polyandry in the face of putative direct costs (Evans and Simmons 2008; Klemme et al. 2014). Our model confirms that polyandry cannot evolve solely due to the pure SSS process when multiple mating is costly, irrespective of the form of fertilization and even if the cost is very small, without some additional force (just as costly preferences for displays cannot evolve through basic indirect selection).

Negatively biased mutations in fertilization efficiency have been hypothesized to maintain genetic variance and rescue the SSS process, thereby maintaining nonzero polyandry even when multiple mating is costly (Keller and Reeve 1995). Such rescue can occur for preference and display (Pomiankowski et al. 1991), as illustrated by our mate-choice model. However, our SSS model shows that, contrary to Keller and Reeve's (1995) key suggestion, the same rescue does not necessarily occur for polyandry and fertilization efficiency. Even with a very small cost of polyandry and strong negatively biased mutations in *F*, the mean value of polyandry maintained was often only marginally higher than mutation-selection balance.

Some degree of evolution and maintenance of costly polyandry arose given relatively extreme parameterization of our SSS model, such as “winner-takes-all” fertilization or very high mutation rates plus strong negatively biased mutations (Figs. 5G and 7C, D) and, to a lesser extent, numerous loci per trait (Fig. 7A, B), creating high standing genetic variation. There is therefore scope for some degree of costly polyandry to be maintained via indirect selection stemming from postcopulatory competition over fertilization, provided that high genetic variance in *F* is somehow maintained and the female's mate with the highest *F* gains highly disproportionate paternity. The question, therefore, is whether such high additive genetic variance in fertilization efficiency and/or disproportionate fertilization success exist in nature.

Although substantial additive genetic variance has been estimated in sperm traits that are hypothesized to influence sperm competitiveness, additive genetic variance and heritability in fertilization success itself have proved hard to detect (Simmons and Moore 2009; Tregenza et al. 2009; Dowling et al. 2010; but see Konior et al. 2005). Detection is impeded by the relative nature of fertilization success (given a random sample of male competitors), and by the discrepancy between observed paternity success and underlying fertilization success due to differential embryo viability caused by sire effects (García-González 2008a,b; García-González and Evans 2011) or male-by-female interactions (Evans and Marshall 2005; Droge-Young et al. 2012).

In many species, fertilization success is highly skewed and does appear to reflect a biased raffle (Sakaluk and Eggert 1996; Simmons and Siva-Jothy 1998; Wedell and Cook 1998; Parker and Pizzari 2010; Simmons 2014). However, “fair-raffle” fertilization also appears to be widespread (Gage and Morrow 2003; Engqvist et al. 2007; Manier et al. 2010; Parker and Pizzari 2010; Simmons 2014). Furthermore, many such examples concern insects with limited sperm storage capacity, and “fair-raffle” fertilization may be even more likely when sperm storage is less constrained, such as in many vertebrates and external fertilizers (Parker and Pizzari 2010). Our models suggest that the pure SSS process is unlikely to drive the evolution of polyandry in such systems.

Logic suggests that the SSS process cannot work if male fertilization efficiency is maternally inherited (Pizzari and Birkhead 2002; Evans and Simmons 2008). Our model suggests that the pure SSS process might not generally be a strong evolutionary force underlying polyandry even given simple biparental autosomal inheritance, at least if polyandry incurs any direct cost and if post-copulatory competition for fertilization results in some degree of shared paternity. Our model therefore concurs with the broader theoretical and empirical view that indirect selection on mating strategies might generally be weak, and hence play a relatively minor role in driving evolutionary dynamics (Kirkpatrick 1996; Kirkpatrick and Barton 1997; Cameron et al. 2003; Arnqvist and Kirkpatrick 2005; Jones and Ratterman 2009).

### Model Assumptions and Extensions

Our SSS model was designed to capture the pure SSS process as proposed by Keller and Reeve (1995), and consequently makes some strong assumptions. Our basic model assumes that phenotypes are directly determined by entirely additive genetic effects with no explicit environmental components of phenotypic variance, apart from stochasticity introduced by random sampling of males and translation of continuously distributed genetic variation in female tendency for polyandry into a discrete number of mates. This is unrealistic; life-history traits often show high environmental variance and low heritability (Houle 1992). Introducing additional environmental variance would presumably further weaken the genetic correlation between female and male traits, further diminishing coevolution. Indeed, coevolution scarcely occurred in our SSS model that included male precedence in fertilization according to random mating order and corresponding nongenetic variance. Although fertilization is sometimes independent of mating order (e.g., Zeh and Zeh 1994; Engqvist et al. 2007), some form of precedence occurs widely in species with internal fertilization (Parker 1970; Birkhead and Hunter 1990; Watson 1991; Lacey et al. 1997; Price et al. 1999; Kraaijeveld-Smit et al. 2002; Pischedda and Rice 2012), further challenging the hypothesis that the pure SSS process could, in general, drive evolution of polyandry.

In our SSS model, the degree of precedence could not evolve. However, given the variety of forms of precedence observed in nature, different strategies are likely to have evolved to ensure precedence and/or favorable mating order. An interesting model extension would therefore be to allow mating order to have a genetic basis and hence to evolve, potentially encompassing trade-offs between male efficiency in ensuring favorable mating order and other factors influencing fertilization efficiency.

Keller and Reeve (1995) did not explicitly discuss the relationship between relative male fertilization efficiency and realized fertilization success achieved under postcopulatory competition, meaning that the sensitivity of the SSS process to this relationship has not been highlighted. Keller and Reeve (1995) implicitly suggest a “winner-takes-all” scenario in the context of a simple single-locus biallelic situation, but this might not be generally appropriate. The degree to which the form of fertilization could coevolve with polyandry also merits future attention. For example, our model implies that by ensuring “fair-raffle” rather than “winner-takes-all” fertilization, females could potentially impede runaway evolution of polyandry.

The pure SSS hypothesis also assumes no preexisting genetic covariances between fertilization efficiency and any other components of male or female fitness. Although this could be viewed as a strength, it is also a limitation if other covariances do in fact exist. Indirect selection on polyandry could potentially be facilitated (or hindered) by complex genetic covariances between multiple different traits. For example, we considered *F* as a single trait, but fertilization efficiency often results from multiple interacting traits and postcopulatory processes, including sperm quantity, viability, and displacement ability (Gomendio and Roldan 1993; Keller and Reeve 1995; Snook 2005; Pizzari and Parker 2009; Simmons and Moore 2009). These traits might trade-off and experience divergent selection, potentially helping to maintain genetic variance in overall fertilization efficiency and allowing for complex multiple genetic covariances and coevolutionary dynamics between different male traits and females strategies (Parker 1990; Parker and Pizzari 2010; Engqvist 2012; Alonzo and Pizzari 2013).

Various extensions and variations on the pure SSS hypothesis have been proposed that invoke additional genetic covariances. Most pertinently, the “good-sperm” hypothesis (Yasui 1997; Petrie and Kempenaers 1998), in analogy with the “good-genes” hypothesis for evolution of female preference (Pomiankowski 1987a; Iwasa et al. 1991; Rowe and Houle 1996; Mead and Arnold 2004; Kokko et al. 2006), assumes a priori existence of positive genetic covariance between fertilization efficiency and “viability.” Models suggest that such pleiotropy can facilitate evolution of polyandry (Yasui 1997), but do not explain why the underlying positive covariance exists in the first place. Indeed, empirical evidence of positive genetic covariance between fertilization efficiency and viability, or between polyandry and offspring fitness, remains scarce and contradictory (Simmons and Kotiaho 2002; Hosken et al. 2003; Simmons 2005; García-González and Simmons 2011; Reid and Sardell 2012; Sardell et al. 2012; Slatyer et al. 2012).

Furthermore, fertilization efficiency could positively covary with male attractiveness, especially if both traits were condition dependent, creating positive genetic covariance between pre- and postcopulatory male traits (Birkhead and Pizzari 2002; Kvarnemo and Simmons 2013). Available evidence is inconclusive: positive genetic correlations between male attractiveness and fertilization efficiency have sometimes been observed (Evans et al. 2003; Locatello et al. 2006; Janhunen et al. 2009; Navara et al. 2012), and sometimes not (Birkhead et al. 1997; Pizzari et al. 2004; Evans 2010; Simmons et al. 2010; Engqvist 2011; Mautz et al. 2013). In addition males, rather than solely females, might influence the degree of polyandry, and male and female tendencies for multiple mating might potentially be correlated (Halliday and Arnold 1987; Forstmeier et al. 2011). It therefore remains possible that the SSS process could act alongside other mechanisms of indirect selection. Genetically explicit quantitative theory that considers evolution of polyandry through indirect selection given multidimensional trait space is now required.

## Data Archiving

The doi for our data is 10.5061/dryad.2tr22.

## Supporting Information

Table S1 Summary of variables and parameter values used in analogous models of coevolution of preference (*P*) and display (*D*, mate-choice model, MCM) and polyandry (*Py*) and fertilization efficiency (*F*, SSS model, SSSM). **Figure S1**. Direct selection functions. **Figure S2**. Precedence in fertilization functions. **Figure S3**. Initial allelic and phenotypic distributions. **Figure S4**. Genetic variances and covariances in female and male traits in the absence of direct selection. **Figure S5**. Genetic variances and covariances in female and male traits in the presence of direct selection. **Figure S6**. Genetic variances and covariances in female and male traits in the presence of direct selection and negatively biased mutations in the male trait. **Figure S7**. Genetic correlations between female and male traits in the presence of direct selection and negatively biased mutations on the male trait. **Figure S8**. Effect of the strength of paternity bias (α) linking fertilization efficiency (*F*) to realized fertilization success on coevolution between polyandry (*Py*) and *F* in the absence of direct selection. **Figure S9**. Combined effect of the strength of paternity bias (α) and negatively biased mutations (*m*′) on the evolution of polyandry in the presence of direct selection. **Figure S10**. Genetic variances and covariances in polyandry and fertilization efficiency for different form of fertilization (α). **Figure S11**. Genetic variances and covariances in polyandry and fertilization efficiency in the presence of male precedence. **Figure S12**. Sensitivity to the number of loci (*L*). **Figure S13**. Genetic variances and covariances in preference and display for different numbers of loci. **Figure S14**. Genetic variances and covariances in polyandry and fertilization efficiency for different numbers of loci. **Figure S15**. Sensitivity to mutation rate (μ). **Figure S16**. Genetic variances and covariances in female preference and male display for different mutation rates. **Figure S17**. Genetic variances and covariances in polyandry and fertilization efficiency for different mutation rates. **Figure S18**. Sensitivity to the magnitude of the mutational effect, σ*^2^_m_*. **Figure S19**. Effect of the magnitude of the mutational effect, σ*^2^_m_*, on the female traits mean phenotypes. **Figure S20**. Genetic variances and covariances in preference and display for different magnitude of the mutational effect. **Figure S21**. Genetic variances and covariances in polyandry and fertilization efficiency for different magnitude of the mutational effect.
