# Supplementary material for: Evolution of female multiple mating: A quantitative model of the “sexually selected sperm” hypothesis
Source: Evolution. 2014 Nov 28;69(1):39–58. doi: 10.1111/evo.12550 (PMC4312924; doi:10.1111/evo.12550)
Supplement: Table S1 — Summary of variables and parameter values used in analogous models of coevolution of preference (P) and display (D, mate-choice model, MCM) and polyandry (Py) and fertilization efficiency (F, SSS model, SSSM). Figure S1. Direct selection functions. Figure S2. Precedence in fertilization functions. Figure S3. Initial allelic and phenotypic distributions. Figure S4. Genetic variances and covariances in female and male traits in the absence of direct selection. Figure S5. Genetic variances and covariances in female and male traits in the presence of direct selection. Figure S6. Genetic variances and covariances in female and male traits in the presence of direct selection and negatively biased mutations in the male trait. Figure S7. Genetic correlations between female and male traits in the presence of direct selection and negatively biased mutations on the male trait. Figure S8. Effect of the strength of paternity bias (α) linking fertilization efficiency (F) to realized fertilization success on coevolution between polyandry (Py) and F in the absence of direct selection. Figure S9. Combined effect of the strength of paternity bias (α) and negatively biased mutations (m′) on the evolution of polyandry in the presence of direct selection. Figure S10. Genetic variances and covariances in polyandry and fertilization efficiency for different form of fertilization (α). Figure S11. Genetic variances and covariances in polyandry and fertilization efficiency in the presence of male precedence. Figure S12. Sensitivity to the number of loci (L). Figure S13. Genetic variances and covariances in preference and display for different numbers of loci. Figure S14. Genetic variances and covariances in polyandry and fertilization efficiency for different numbers of loci. Figure S15. Sensitivity to mutation rate (μ). Figure S16. Genetic variances and covariances in female preference and male display for different mutation rates. Figure S17. Genetic variances and covariances in polyandry [file evo0069-0039-sd1.docx]

**EVOLUTION OF FEMALE MULTIPLE MATING: A QUANTITATIVE MODEL OF THE “SEXUALLY-SELECTED SPERM” HYPOTHESIS**

Greta Bocedi and Jane M. Reid

*Supporting Information*

**Table S1.** Summary of variables and parameter values used in analogous models of co-evolution of preference (*P*) and display (*D*, mate-choice model, MCM) and polyandry (*Py*) and fertilization efficiency (*F*, SSS model, SSSM). See Supplementary Information for sensitivity analyses.

|  | **Variables** | **Description** | **Parameter values** |
| --- | --- | --- | --- |
|  | *K* | carrying capacity | 1000 individuals |
|  | *R* | mean fecundity | 4 |
|  | *L* | number of diploid loci for each trait | 20 |
| **Mutations** | *µ* | mutation probability (per allele per generation) | 0.001 |
|  | *m* | mean mutational effect | 0.0 |
|  | *m’* | mean mutational effect for male traits (*D* or *F*) in the case of negatively biased mutations | -0.05, -0.10, -0.15, -0.20, -0.25, -0.3, -0.35 |
|  | *σ^2^_m_* | variance in mutational effects | = (1.0/2*L*)*0.05 |
| **Traits** | *gP* | female preference for male display (genotypic value) |  |
|  | *gD* | male display (genotypic value) |  |
|  | *P* | female preference for male display (phenotypic value) | = $\left\{ \begin{matrix} 0 & \mathrm{for} & gP<0 \\ gP & \mathrm{for} & gP \geq0 \end{matrix} \right.$ |
|  | *D* | male display (phenotypic value) | = *gD* |
|  | *gPy* | female tendency for polyandry (genotypic value) |  |
|  | *gF* | male fertilization efficiency (genotypic value) |  |
|  | *Py* | female tendency for polyandry (phenotypic value) | = $\left\{ \begin{matrix} 0 & \mathrm{for} & gPy<0 \\ gPy & \mathrm{for} & gPy \geq0 \end{matrix} \right.$ |
|  | *F* | male fertilization efficiency (phenotypic value) | = *gF* |
| **Trait Initialisation** | *µ_P,0_* = *µ_Py,0_* | initial phenotypic mean for female preference (MCM) and tendency for polyandry (SSSM) | 1.0 |
|  | *µ_D,0_* = *µ_F,0_* | initial phenotypic mean for male display (MCM) and fertilization efficiency (SSSM) | 3.0 |
|  | *σ^2^_P,0_* = *σ^2^_D,0_* = *σ^2^_Py,0_* = *σ^2^_F,0_* | initial phenotypic variance for the four traits | 1.0 |
|  | *µ_aP,0_* = *µ_aPy,0_* | initial allelic mean for female preference (MCM) and tendency for polyandry (SSSM) | = *µ_P,0_* / 2*L* = *µ_Py,0_* / 2*L* |
|  | *µ_aD,0_* = *µ_aF,0_* | initial allelic mean for male display (MCM) and fertilization efficiency (SSSM) | = *µ_D,0_* / 2*L* = *µ_F,0_* / 2*L* |
|  | *σ^2^_aP,0_* = *σ^2^_aD,0_* = *σ^2^_aPy,0_* = *σ^2^_aF,0_* | initial allelic variance for the four traits | = *σ^2^_P,0_* / 2*L* = *σ^2^_D,0_* / 2*L* =  *σ^2^_Py,0_* / 2*L* = *σ^2^_F,0_* / 2*L* |
| **Costs** | *θ_P_* = *θ_Py_* | naturally selected optimum for preference (MCM) and tendency for polyandry (SSSM) | 0.0 |
|  | *θ_D_* = *θ_F_* | naturally selected optimum for display (MCM) and fertilization efficiency (SSSM) | 3.0 |
|  | *ω^2^_P_* = *ω^2^_Py_* | strength of stabilising natural selection for preference (MCM) and tendency for polyandry (SSSM) | 1.0, 25.0, 50.0, 100.0, 200.0, 400.0 (Fig. S1) |
|  | *ω^2^_D_* = *ω^2^_F_* | strength of stabilising natural selection for display (MCM) and fertilization efficiency (SSSM) | NA, 1.0 |
|  | *Nmales_I_* | number of randomly selected males (MCM) | 5 |
|  | *α* | strength of paternity bias | 0.5, 1, 2, 5, 10 (the latter represents ’winner-takes-all’) |
|  | *β* | strength of male precedence (SSSM) | 0, 0.5, 1, 2, 10 (the latter represents complete first male precedence) (Fig. S2) |

**Figure S1. Direct selection functions.**

Individual viability *v_i_* when the trait *t* is under direct selection towards the naturally selected optimum *θ_t_* = 0. Different values of *ω^2^_t_* represent different strengths of selection (eqn. 5).

**Figure S2. Precedence in fertilization functions.**

Male precedence in fertilization. Relationships between male rank (mating order) and probability of fertilizing each of a female’s eggs (eqn. 8). *β* = 10 represents the extreme case of first-male precedence where the first male sires all the offspring, while *β* = 0 correspond to no precedence. The first male to mate is assigned rank = 0.

**Figure S3. Initial allelic and phenotypic distributions.**

**A:** Initial allelic and phenotypic distributions for the female traits, preference (*P*) or tendency for polyandry (*Py*). **B:** Initial allelic and phenotypic distributions for the male traits, display (*D*) or fertilization efficiency (*F*). Dashed lines represent the initial means.

**Figure S4. Genetic variances and covariances in female and male traits in the absence of direct selection.**

**A:** Mean genetic variance in preference (*gP*) and display (*gD*), and genetic covariance between the two. **B:** Mean genetic variance in polyandry (*gPy*) and fertilization efficiency (*gF*), and genetic covariance between the two. Data are plotted every 50 generations to generation 1000 and every 100 generations thereafter. **C-D:** same as A-B but showing the first 100 generations at 5 generations interval. Mean values (black lines) ± standard deviation (grey shade) were calculated across 50 replicate simulations.

**Figure S5.** **Genetic variances and covariances in female and male traits in the presence of direct selection.**

**A:** Mean genetic variance in preference (*gP*) and display (*gD*), and genetic covariance between the two, for different strengths of direct selection on preference (*ω^2^_P_*). **B:** Mean genetic variance in polyandry (*gPy*) and fertilization efficiency (*gF*), and genetic covariance between the two, for different strengths of direct selection on polyandry (*ω^2^_Py_*). Data are presented every 50 generations until generation 1000 and every 100 generations thereafter. **C-D:** same as A-B but showing the first 100 generations at an interval of 5 generations. All mean values were calculated across 50 replicate simulations.

**Figure S6.** **Genetic variances and covariances in female and male traits in the presence of direct selection and negatively biased mutations in the male trait.**

**Left panels:** Mean genetic variance in *gP* and *gPy*. **Centre panels:** Mean genetic variance in *gD* and *gF*. **Right panels:** genetic covariance between the female and male traits. Mean values were calculated across 50 replicate simulations for three different levels of direct selection on the female traits (*ω^2^_P_* and *ω^2^_Py_*) and plotted every 50 generations until generation 1000 and every100 generations thereafter.

**Figure S7. Genetic correlations between female and male traits in the presence of direct selection and negatively biased mutations on the male trait.**

Genetic correlations between preference (*P*) and display (*D*, left panels) and between polyandry (*Py*) and fertilization efficiency (*F*, right panels), for three levels of direct selection on *P* and *Py* (*ω^2^_P_* and *ω^2^_Py_*). For all simulations *ω^2^_D_* = *ω^2^_F_* = 1.0. Mean values were calculated across 50 replicate simulations every 50 generations until generation 1000 and every 100 generations thereafter.

**Figure S8. Effect of the strength of paternity bias (*α*) linking fertilization efficiency (*F*) to realised fertilization success on co-evolution between polyandry (*Py*) and *F* in the absence of direct selection.** Overall mean phenotypic trait values (black lines) and mean values for individual replicate simulations (grey lines). All means are averaged over 50 replicate simulations and plotted every 50 generations until generation 1000 and 100 generations thereafter.

**Figure S9. Combined effect of the strength of paternity bias (*α*) and negatively-biased mutations (*m’*) on the evolution of polyandry in the presence of direct selection.** Mean polyandry (*Py*) at generation 5000, for different strengths of paternity bias (α) linking fertilization efficiency (*F*) to realised fertilization success and different strengths of negatively-biased mutations on *F*. **A:** *ω^2^_Py_* = 100. **B:** *ω^2^_Py_* = 200. **C:** *ω^2^_Py_* = 400. Data are presented as medians (solid bands), first and third quartiles (box limits) and approximately twice the standard deviation (whiskers) over 50 replicate simulations.

**Figure S10. Genetic variances and covariances in polyandry and fertilization efficiency for different form of fertilization (*α*).**

Genetic variances in polyandry (*Py*) and fertilization efficiency (*F*), and genetic covariance between the two, for different relationships between fertilization efficiency and realised fertilization success (*α*). **A:** No direct selection on *Py* and *F*. **B:** Direct selection on *Py*, *ω^2^_Py_* = 400.0, and *F*, *ω^2^_F_* = 1.0. **C:** same as in B when, additionally to direct selection, negatively-biased mutations are applied to *F*: *m’* = -0.35. Mean values were calculated across 50 replicate simulations every 50 generations until generation 1000 and every 100 generations thereafter.

**Figure S11. Genetic variances and covariances in polyandry and fertilization efficiency in the presence of male precedence.**

Genetic variances in polyandry (*Py*) and fertilization efficiency (*F*), and genetic covariance between the two, for different degrees of male precedence in fertilization (*β*). **A:** No direct selection on *Py* and *F*. **B:** Direct selection on *Py*, *ω^2^_Py_* = 400.0, and *F*, *ω^2^_F_* = 1.0. **C:** same as in B when, additionally to direct selection, negatively biased mutations are applied to the male trait: *m’* = -0.35. **D:** same as in C when fertilization is biased towards males with higher *F* (*α* = 5). Mean values were calculated across 50 replicate simulations every 50 generations until generation 1000 and every 100 generations thereafter.

**SENSITIVITY TO THE NUMBER OF LOCI**

We tested the sensitivity of the mate-choice and sexually-selected sperm models to the number of loci, *L*, underlying each trait’s genotypic value. We ran simulations with the following parameters combinations (for a complete description of all model variables and parameters see Table 1 in the main text):

| **Variables** | **Parameter values** |
| --- | --- |
| ***L*** | **1, 20 (default), 40, 100** |
| *µ* | 0.001 |
| *m* | 0.0 |
| *σ^2^_m_* | = (1.0/2*L*)*0.05 |
| *θ_P_* = *θ_Py_* | 0.0 |
| *θ_D_* = *θ_F_* | 3.0 |
| *ω^2^_P_* = *ω^2^_Py_* | 100.0, 200.0, 400.0 |
| *ω^2^_D_* = *ω^2^_F_* | 1.0 |
| *β* | 0 |

Note that the initial allelic values (Table 1) are scaled according to *L* so that the initial genotypic and phenotypic variances are constant for all simulations. The mutational effects are also scaled according to *L*, hence the higher the number of loci the smaller the per locus mutational change.

**Figure S12: Sensitivity to the number of loci (*L*).**

Mean phenotypic values of preference (*P*) and display (*D*) and of polyandry (*Py*) and fertilization efficiency (*F*), when modelled with different numbers of loci (*L*) for each trait. **A:** No direct selection applied to any of the traits. **B:** Direct selection on both traits: *ω^2^_P_* = *ω^2^_Py_* = 100.0. **C:** *ω^2^_P_* = *ω^2^_Py_* = 200.0. **D:** *ω^2^_P_* = *ω^2^_Py_* = 400.0. All data are plotted every 50 generations until generation 1000 and 100 generations thereafter. All means are averaged over 50 replicate simulations.

Dashed lines indicate the traits’ optima. Grey circles represent the simulation starting values.

**Figure S13: Genetic variances and covariances in preference and display for different numbers of loci.**

Mean genetic variance in preference (*gP*) and display (*gD*), and covariance between the two, for models differing in the number of loci (*L*). **A-B:** No direct selection applied to any of the traits. **C-D:** Direct selection on both traits: *ω^2^_P_* = 200.0, *ω^2^_D_* = 1.0. All data are averaged over 50 replicates and presented either every 50 generations until generation 1000 and every 100 generations thereafter (A and C) or every 5 generations (B and D).

**Figure S14: Genetic variances and covariances in polyandry and fertilization efficiency for different numbers of loci.**

Mean genetic variance in polyandry (*gPy*) and fertilization efficiency (*gF*), and covariance between the two, for models differing in the number of loci (*L*). **A-B:** no direct selection applied to any of the traits. **C-D:** direct selection on both traits: *ω^2^_P_* = 200.0, *ω^2^_D_* = 1.0. All data are averaged over 50 replicates and presented either every 50 generations until generation 1000 and every 100 generations thereafter (A and C) or every 5 generations (B and D).

**SENSITIVITY TO MUTATION RATE, *µ***

To test the sensitivity of the mate-choice and sexually-selected sperm models to the mutation rate at each allele/generation, *µ*, we ran simulations with the following parameters:

| **Variables** | **Parameter values** |
| --- | --- |
| *L* | 20 |
| ***µ*** | **10^-4^, 10^-3^ (default), 10^-2^** |
| *m* | 0.0 |
| *m’* | -0.35 |
| *σ^2^_m_* | = (1.0/2*L*)*0.05 |
| *θ_P_* = *θ_Py_* | 0.0 |
| *θ_D_* = *θ_F_* | 3.0 |
| *ω^2^_P_* = *ω^2^_Py_* | 200.0 |
| *ω^2^_D_* = *ω^2^_F_* | 1.0 |
| *β* | 0 |

**Figure S15: Sensitivity to mutation rate (*µ*)**

Effect of different mutation rates (*µ*) on the mean phenotypic values of preference (*P*) and display (*D*) and of polyandry (*Py*) and fertilization efficiency (*F*). **A:** no direct selection applied to any of the traits. **B:** direct selection on both traits: *ω^2^_P_* = *ω^2^_Py_* = 200.0 and *ω^2^_D_* = *ω^2^_F_* = 1.0. **C:** negative mutation bias on *D* and *F* (*m’* = -0.35); direct selection is the same as in B. For all simulations the mutation size is *σ^2^_m_* = (1.0/2L)*0.05. Dashed lines indicate the traits’ optima. Grey dots indicate the simulation starting values. All data are averaged over 50 replicates and plotted every 50 generations until generation 1000 and every 100 generations thereafter.

**Figure S16: Genetic variances and covariances in female preference and male display for different mutation rates.**

Mean genetic variance in preference (*gP*) and display (*gD*), and covariance between the two, for models differing in the mutation rate (*µ*). **A:** No direct selection applied to any of the traits. **B:** Direct selection on both traits: *ω^2^_P_* = 200.0, *ω^2^_D_* = 1.0. **C:** negative mutation bias on *D* (*m’* = -0.35); direct selection is the same as in B. Data are averaged over 50 replicates and plotted every 50 generations until generation 1000 and every 100 generations thereafter.

**Figure S17: Genetic variances and covariances in polyandry and fertilization efficiency for different mutation rates.**

Mean genetic variance in polyandry (*gPy*) and fertilization efficiency (*gF*), and covariance between the two, for models differing in the mutation rate (*µ*). **A:** no direct selection applied to any of the traits. **B:** direct selection on both traits: *ω^2^_Py_* = 200.0, *ω^2^_F_* = 1.0. **C:** negative mutation bias on *F* (*m’* = -0.35); direct selection is the same as in B. Data are averaged over 50 replicates and plotted every 50 generations until generation 1000 and every 100 generations thereafter.

**SENSITIVITY TO THE MAGNITUDE OF THE MUTATIONAL EFFECT, *σ^2^_m_***

A final analysis was conducted to investigate the sensitivity of the mate-choice and sexually-selected sperm models to the magnitude of the mutational effect, *σ^2^_m_*. The parameters explored are reported below.

| **Variables** | **Parameter values** |
| --- | --- |
| *L* | 20 |
| *µ* | 10^-3^ |
| *m* | 0.0 |
| *m’* | -0.35 |
| ***σ^2^_m_*** | **= (1.0/2*L*)*0.2**  **= (1.0/2*L*)*0.05**  **= (1.0/2*L*)*0.01** |
| *θ_P_* = *θ_Py_* | 0.0 |
| *θ_D_* = *θ_F_* | 3.0 |
| *ω^2^_P_* = *ω^2^_Py_* | 200.0 |
| *ω^2^_D_* = *ω^2^_F_* | 1.0 |
| *β* | 0 |

**Figure S18: Sensitivity to the magnitude of the mutational effect, *σ^2^_m_*.**

Effect of varying the variance of the distribution of mutational effects (*σ^2^_m_*) on the mean phenotypic values of preference (*P*) and display (*D*) and of polyandry (*Py*) and fertilization efficiency (*F*). **A:** no direct selection applied to any of the traits. **B:** direct selection on both traits: *ω^2^_P_* = *ω^2^_Py_* = 200.0 and *ω^2^_D_* = *ω^2^_F_* = 1.0. **C:** negative mutation bias on *D* and *F* (*m’* = -0.35); direct selection is the same as in B. For all simulations *µ* = 0.001. Dashed lines indicate the traits’ optima. Grey circles indicate the simulation starting values. Data are averaged over 50 replicates and plotted every 50 generations until generation 1000 and every 100 generations thereafter.

**Figure S19: Effect of the magnitude of the mutational effect, *σ^2^_m_*, on the female traits mean phenotypes.**

Effect of different variance of the mutational effects distribution (*σ^2^_m_*) on the mean phenotypic values for preference (*P*) and display (*D*) and for polyandry (*Py*) and fertilization efficiency (*F*) (black boxes), and neutral traits subject only to mutations or to the same costs, at generation 5000. **A:** no direct selection applied to any of the traits. **B:** direct selection on both traits: *ω^2^_P_* = *ω^2^_Py_* = 200.0 and *ω^2^_D_* = *ω^2^_F_* = 1.0. **C:** negative mutation bias on *D* and *F* (*m’* = -0.35); direct selection is the same as in B. Data are described as medians (solid bands), first and third quartiles (box limits), approximately twice the standard deviation (whiskers) and means (diamonds).

**Figure S20: Genetic variances and covariances in preference and display for different magnitude of the mutational effect.**

Mean genetic variance in preference (*gP*) and display (*gD*), and covariance between the two, for models differing in the variance of the mutational effects distribution (*σ^2^_m_*). **A:** no direct selection. **B:** direct selection on both traits: *ω^2^_P_* = 200.0, *ω^2^_D_* = 1.0. **C:** negative mutation bias on *D* (*m’* = -0.35); direct selection is the same as in B. Data are averaged over 50 replicates and presented every 50 generations until generation 1000 and every 100 generations thereafter.

**Figure S21: Genetic variances and covariances in polyandry and fertilization efficiency for different magnitude of the mutational effect.**

Mean genetic variance in polyandry (*gPy*) and fertilization efficiency (*gF*), and covariance between the two, for models differing in the variance of the mutational effects distribution (*σ^2^_m_*). **A:** no direct selection. **B:** direct selection on both traits: *ω^2^_Py_* = 200.0, *ω^2^_F_* = 1.0. **C:** negative mutation bias on *F* (*m’* = -0.35); direct selection is the same as in B. Data are averaged over 50 replicates and presented every 50 generations until generation 1000 and every 100 generations thereafter.
